# Experimental PVC Material Challenge in Subjects with Occupational PVC Exposure

**DOI:** 10.1289/ehp.8965

**Published:** 2006-05-18

**Authors:** Anneli Tuomainen, Harri Stark, Markku Seuri, Maija-Riitta Hirvonen, Markku Linnainmaa, Anne Sieppi, Hannu Tukiainen

**Affiliations:** 1 Technology Centre Teknia Ltd., Kuopio, Finland; 2 Department of Respiratory Medicine, Kuopio University Hospital, Kuopio, Finland; 3 Occupational Health Services, Atria Ltd., Nurmo, Finland; 4 Department of Environmental Health, National Public Health Institute, Kuopio, Finland; 5 Department of Occupational Hygiene and Toxicology, Finnish Institute of Occupational Health, Kuopio, Finland; 6 Medivire Occupational Health Centre, Kuopio, Finland

**Keywords:** asthma, chamber exposure, cytokine, nitric oxide, PVC flooring material

## Abstract

**Background:**

Polyvinyl chloride (PVC) materials have been linked to asthma in several epidemiologic studies, but the possible causal factors remain unknown.

**Participants:**

We challenged 10 subjects experimentally to degraded PVC products under controlled conditions. All of the subjects had previously experienced respiratory symptoms suspected to be caused by this kind of exposure in their work place. Five subjects had doctor-diagnosed asthma.

**Methods:**

The subjects were exposed to degraded PVC material in an exposure chamber; a challenge with ceramic tile was used as the control test. We followed exhaled nitric oxide, nasal NO, lung functions, cytokines [tumor necrosis factor-α (TNF-α), interleukin-4 (IL-4), IL-6, and IL-12] and NO in nasal lavage fluid (NAL) during and after the exposures. We also measured 2-ethylhexanol in exhaled breath samples and NAL.

**Results:**

On the morning after the PVC exposure, subjects reported respiratory tract symptoms significantly more often than they did after the control test (50% vs. 0%, respectively; *p* = 0.029; *n* = 10). We did not detect any changes in lung functions or levels of exhaled NO, nasal NO, or NO in NAL after PVC challenge compared with the control test. Cytokine levels increased after both exposures, with no statistically significant difference between situations. All of the exhaled breath samples collected during the PVC exposure contained 2-ethylhexanol.

**Conclusions:**

PVC flooring challenge can evoke respiratory tract symptoms in exposed subjects. Our results do not support the hypothesis that PVC materials themselves evoke immediate asthmatic reactions. The chamber test used is well suited to this type of exposure study.

The incidence of asthma and allergy has increased during the past decades; this increase is considered to be caused by environmental exposure rather than genetic factors (Beasley et al. 2003; Selgrade et al. 2006). However, the mechanisms activated in exposed respiratory cells and tissues, as well as the causative particulate constituents and sources, remain to be clarified. In several epidemiologic studies the use of polyvinyl chloride (PVC) materials in indoor environments has been associated with an increased risk of asthma and allergy. It has been proposed that the plasticizer used in PVC carpets, diethylhexyl phthalate (DEHP), which binds onto the surface of building materials, may lead to the formation of substantial amounts of inhalable particles contaminated with agents of PVC origin (Øie et al. 1997). Recently, DEHP concentrations were found to be higher in buildings erected before 1960 (Bornehag et al. 2005a). This could reflect the higher fractional concentrations in older products or the higher emission rates as these products degrade.

Thus, plastic building materials are potential chemical emission sources in indoor air. These emissions can cause inflammation and subsequently lead to an increased risk of asthma. This hypothesis is supported by studies on nasal, conjunctival, and asthmatic symptoms in relation to building dampness and the degradation of PVC flooring material (Norbäck et al. 2000; Wieslander et al.1999). These studies have shown that the degradation of DEHP from PVC flooring evokes conjunctival and nasal irritation and increases asthma-like symptoms in the exposed subjects. Furthermore, one PVC degradation product, 2-ethylhexanol, was detected in indoor air samples. Studies in young children have also revealed an association between the chemical emissions of PVC materials into indoor air and adverse respiratory effects (Jaakkola et al. 1999, 2000). In addition, Bornehag et al. (2004) reported that there was a dose–response relationship between asthma prevalence in children and the concentrations of DEHP in settled dust in their environments. The combination of water leakage and PVC as flooring material was also shown to be associated with a higher prevalence of airway, nasal, and dermal symptoms in exposed children (Bornehag et al. 2005b).

Jaakkola et al. (1999) and Øie et al. (1997) proposed that phthalate esters act as either allergens or adjuvants; although there does seem to be a clear association between health problems and PVC materials, the etiologic factors behind this link are still unclear. In the present study, 10 subjects exposed to degrading PCV products at their workplace were challenged by PVC under controlled conditions. We have previously reported the renovation work being carried out in the building (Tuomainen et al. 2004).

## Materials and Methods

### Subjects

Ten volunteer subjects (seven males, three females) provided their written informed consent prior to the study. The mean age of the subjects was 43.2 years (range, 28–53 years). There were two current smokers and two ex-smokers among the subjects, and the others had never smoked. Smoking was prohibited during the study periods. Five subjects had doctor-diagnosed asthma, three of them having received this diagnosis during occupational exposure to degraded PVC flooring material. The subjects were asked not to use inhaled steroids, long-acting β_2_-adrenoreceptor agonists, leukotriene receptor antagonists, or mast cell stabilizers for 3 days before the challenges or short-acting β_2_-adrenoreceptor agonists for 6 hr before the challenges. The five non-asthmatic subjects also presented with a variety of symptoms: upper and lower respiratory tract symptoms, conjunctival irritation, and eczema. All of the subjects worked in a building where the initial indoor air evaluations were made in 1997; the source of the symptoms in the occupants was later traced to degrading PVC flooring materials, as described previously (Tuomainen et al. 2004).

The study was approved by the ethics committee of Helsinki University Hospital, which guarantees that approved studies fulfill the requirements of international regulations. We conducted this study according to principles of the Declaration of Helsinki (World Medical Association 2004).

### Chamber exposure tests

The exposure chamber (1.2 × 1.2 × 2.2 m) was constructed of stainless steel and was equipped with a small window. The chamber had separate ventilation that was adjusted so the carbon dioxide concentration stayed < 1,000 ppm during the exposure session. Before conducting the experiments, we tested the chamber and determined that it was sealed properly and clean [total volatile organic compounds (TVOCs) < 10 μg/m^3^]. The material used in the exposure tests was a 1-m^2^ piece of PVC flooring material recently removed from an office where the occupants had suffered respiratory and dermal irritation symptoms related to degrading PVC. The piece of PVC flooring material was replaced every week. During the control tests, we placed unused 1-m^2^ ceramic tiles in the chamber floor. During every PVC exposure test, we measured TVOC and 2-ethylhexanol concentrations in the chamber air (Tuomainen et al. 2004).

Two hours after the beginning of the exposure, we collected and analyzed VOCs from the chamber air and from the exhaled breath into laminate bags as described previously (Tuomainen et al. 2001). Similar laminate bags have been used in collecting air samples such as sulfur oxides, and the bags do not release any emissions themselves. In the chamber, the sample was always collected near the breathing zone of the test subject. We measured concentrations of TVOCs and 2-ethylhexanol in each sample.

The protocol of the chamber challenge tests is shown in Table 1. The challenges lasted 4 hr, during which the subject remained in the chamber. We performed PVC and control exposures in a random order, and the subjects were blinded to the type of exposure. The interval between the two exposures ranged from 3 to 7 weeks.

### Lung function tests

We measured standard spirometric values according to American Thoracic Society guidelines (American Thoracic Society 1991), and we used reference values of Viljanen et al. (1982) for the Finnish population. We recorded spirometric and peak expiratory flow (PEF) values during and after the exposures (Table 1).

### Measurement of exhaled and nasal NO

We measured exhaled nitric oxide with a chemiluminescence analyzer (Sievers Model 280 NOA; Sievers Instruments Inc., Boulder, CO, USA) according to American Thoracic Society recommendations (American Thoracic Society 1999). During measurement of exhaled NO, the subjects performed a slow vital capacity maneuver for 30 sec against a fixed expiratory resistance. We optimized the pressure level during exhalation by following online via the computer screen so that the subjects could achieve a constant flow rate in exhaled air. Exhaled air was led through a non-breathing valve into a Teflon tubing system connected to the analyzer. We performed the recordings using the single-breath program, and observed measurements on the computer screen during the tests. The relative SD between three exhaled samples was expected to be < 10%, and the detection limit for NO was 1 ppb. Measurements were performed in the same laboratory under the same conditions. The chemiluminescence analyzer was calibrated daily by using zero air and a certified concentration of NO.

When measuring nasal NO levels, we used a modification of the fixed-flow exhalation technique of Stark et al. (2005). Two soft well-fitting nose pieces were placed at the entrance of both nostrils. The pieces were attached via a “Y” connector to a two-way valve, and a resistor was placed in the exhalation limb, which required a pressure of 10 cm H_2_O to produce a flow of 100 mL/sec. The subjects inhaled normal room temperature air to total lung capacity (TLC) via their mouths and exhaled nasally, while targeting a flow signal displayed on the computer monitor. The expiration was continued until a steady NO plateau lasting at least 10 sec was reached. The contribution of any oral NO was excluded. The measurement was repeated 3 times, and the mean value was calculated.

The measurement schedule of exhaled and nasal NO during and after the exposures is shown in Table 1.

### Nasal lavage

We collected the nasal lavage fluid (NAL) samples according to the protocol described by Hirvonen et al. (1999) with some modifications. First, 4.5 mL prewarmed Hanks’ balanced salt solution (37°C) was instilled through a heat-softened catheter into the subject’s nostril. During the instillation, the subject held his or her chin down toward the chest and held the catheter in place by pinching the nostrils closed. The cartilaginous bridge of the nose was vibrated with a neonatal percussor (Neo-Cussor, General Physiotherapy Inc., St. Louis, MO, USA) while the fluid was refluxed three times. Subsequently, we repeated the same protocol on the other nostril. The sample was centrifuged (425 × *g*, 10 min) and the cells were resuspended in 2 mL of the supernatant. The remaining cell suspension was incubated for 24 hr at 37°C and then centrifuged (425 × *g*, 10 min). The supernatant and cells were frozen at −70°C.

### Analysis of cytokines, NO, protein, and 2-ethylhexanol in NAL

We analyzed the concentrations of tumor necrosis factor-α (TNF-α), interleukin-4 (IL-4), IL-6, and IL-12 in the NAL supernatant using enzyme-linked immunosorbent assay (ELISA) kits (R&D Systems, Minneapolis, MN, USA). Assays were performed according to the manufacturer’s instructions and analyzed with an ELISA microplate reader (iEMS Reader MF, Labsystems, Helsinki, Finland) at a wavelength of 450 nm by comparing the absorbances of the samples to the standard curve. Each standard and sample was run in duplicate. The concentration of NO in the NAL supernatant was assayed by the Griess reaction as the stable NO oxidation product nitrite (Green et al. 1982) as described in detail by Hirvonen et al. (1999). We collected the NAL samples for cytokine and NO determinations before and immediately after the exposures (Table 1). In addition to exposure tests, we measured cytokine and NO concentrations in NAL after each subject had at least a 1 week holiday from work.

A portion of the NAL was centrifuged at 1,800 rpm (10 min), after which 200 μL was separated into an Eppendorf tube for protein measurement; this sample, as well as the rest of the supernatant, were stored at −18°C before the analysis of proteins and assay of 2-ethylhexanol. We measured the protein content of the samples in a microplate reader using the method of Bradford (1976). 2-Ethylhexanol present in the NAL was extracted with ethyl acetate and analyzed by gas chromatography and mass spectrometry.

### Cytospin

Cytocentrifuge preparations were made from the NAL samples using 100 μL resuspended cell suspension, in which the mucus had been broken by addition of 0.5% dithiothreitol/0.1% bovine serum albumin. The solution was centrifuged, cells were placed on slides, and the slides were stained with May-Grunwald-Giemsa stain (Prat et al. 1993) for the cell differential counts.

### Symptoms related to the exposures

The subjects were asked to report respiratory or any other symptoms during both PVC and control exposure sessions, as well as the morning after the tests. The subjects wrote down their symptoms in the PEF follow-up questionnaire.

### Statistical methods

We evaluated the changes in different parameters during and after the exposures using variance analysis of repeated measures. Logarithmic transformations were performed for data not normally distributed. Between-groups differences were analyzed by the Fisher’s exact test. We used the Pearson’s test to determine correlations, and we compared percentages using the chi-square test. SPSS for Windows 11.5 (SPSS Inc., Chicago, IL, USA) was used in the analysis of the data; *p* < 0.05 was considered statistically significant.

## Results

### TVOC and 2-ethylhexanol concentrations

**Figure 1:**
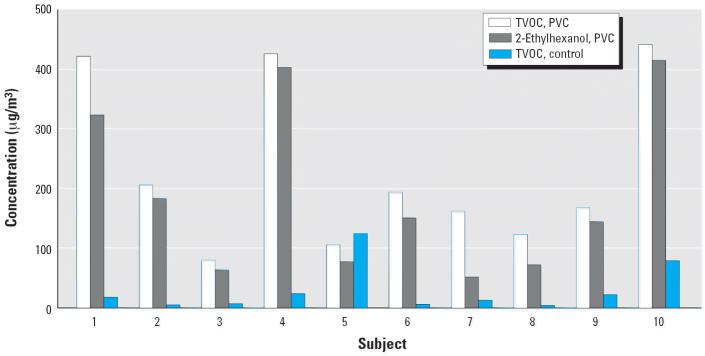
Concentrations of TVOCs and 2-ethylhexanol during the PVC exposure and TVOCs during the control (ceramic tile) test in exposure chamber air.

**Figure 2:**
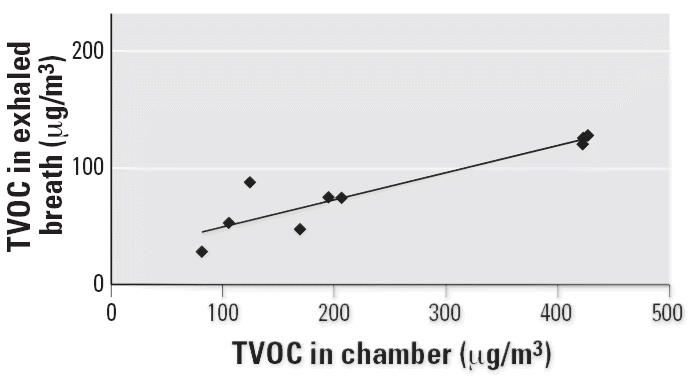
Correlation between TVOCs in exposure chamber air and in exhaled breath samples during exposure to PVC. *R*^2^ = 0.84.

**Figure 3:**
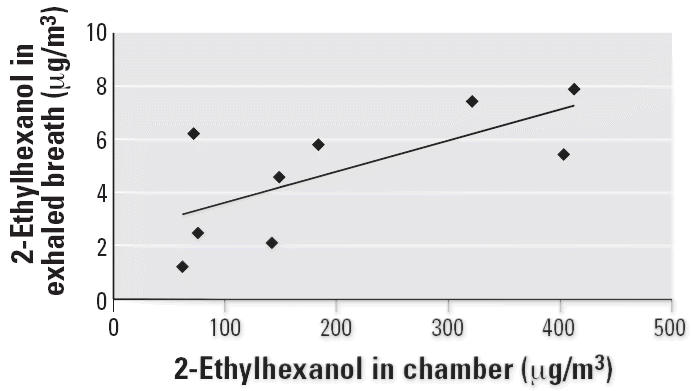
Correlation between 2-ethylhexanol concentrations in exposure chamber air and in exhaled breath samples during exposure to PVC. *R*^2^ = 0.4792.

Concentrations of TVOC and 2-ethylhexanol in the chamber air during the PVC challenges and TVOC during the control tests are shown in Figure 1. During the control exposure, 2-ethylhexanol concentrations were very low in the chamber air (≤ 2.4 μg/m3). The correlation between TVOC levels in exposure chamber air and in exhaled breath samples during the PVC exposure is shown in Figure 2. Figure 3 shows the correlation between concentrations of 2-ethylhexanol in the chamber and in exhaled breath samples during PVC exposure. During the PVC exposure tests, all of the subjects’ exhaled breath samples contained 2-ethylhexanol, with the concentrations ranging from 1.2 to 9.2 μg/m^3^ (mean, 5.2 μg/m^3^).

### Symptoms related to the challenge

The number of subjects reporting respiratory tract symptoms (e.g., nasal symptoms, cough, phlegm) was significantly higher the morning after exposure to the PVC material compared with the morning after control exposure (50% vs. 0%, respectively; *p* = 0.029, *n* = 10). During PVC exposure, subjects experienced respiratory tract and conjunctival symptoms more often than during control challenge, but the differences were not statistically significant (data not shown).

### Lung functions

Before the PVC challenge, the baseline values of forced expiratory volume in 1 sec (FEV_1_) and forced vital capacity (FVC) were 109% and 120%, respectively, of the predicted values. The spirometric values did not change significantly during or after the PVC challenge, nor did we detect any significant differences in lung functions between the PVC and control exposures. The baseline levels of lung functions did not differ significantly in subjects with asthma or in smokers (including current and ex-smokers) compared with the subjects without asthma or nonsmokers (data not shown).

PEF values did not change significantly during or after the challenges, and we found no significant difference in PEF values between PVC and control exposures (data not shown).

### Exhaled and nasal NO

The exhaled NO levels did not change significantly during or after the PVC challenge, and we did not find any significant differences in exhaled NO concentrations between the PVC and control exposures (Table 2). Furthermore, there were no significant changes in nasal NO levels during or after the PVC exposure or any statistically significant differences between the PVC and control challenges (Table 3).

### Cytokines, NO, and 2-ethylhexanol in NAL

The levels of cytokines in NAL (TNF-α, IL-4, IL-6, and IL-12) increased immediately after exposure to either PVC flooring or the control material (Table 4); we found no statistically significant differences in the cytokine concentrations between the challenges. We did not detect any changes in NO concentrations of NAL after the PVC exposure nor did we find any statistically significant difference when compared to the control (Table 4).

The cytokine and NO levels in NAL of samples taken after at least 1 week of vacation did not differ from the baseline levels before the exposures. We did not detect the presence of 2-ethylhexanol in the NAL samples collected after the PVC exposure.

### Cell differential count

Neutrophilic cells dominated the NAL cell profile both before and after both exposures. No changes in the proportions of lymphocytes, neutrophils, or eosinophils were observed in the NAL after the challenges. Moreover, we detected no differences in the changes in the cell counts between the PVC and control challenges (data not shown).

## Discussion

In this study, 10 subjects exposed to degraded PVC flooring material at work were challenged with PVC exposure under controlled conditions. This is the first study to expose subjects to very low concentrations of chemical emissions similar to those found in indoor air conditions.

On the morning after the PVC challenge, the subjects suffered respiratory tract symptoms significantly more often compared with the control exposure. This result is in line with studies in which the subjects experienced similar irritation symptoms during the exposure to damaged PVC products (Bornehag et al. 2005b; Wålinder et al. 1999; Wieslander et al. 1999). According to our results, even minuscule emissions of degraded PVC flooring materials that can be found in indoor air are sufficient to evoke respiratory tract symptoms.

The PVC flooring material challenge did not cause any significant changes in lung functions, exhaled NO, or nasal NO levels compared with the control test. Moreover, there were no individual asthmatic reactions, such as a decrease in FEV_1_, after these short exposure times, although half of the subjects had asthma and their asthma medications had been withdrawn before the study. In occupational exposures, the development of asthma requires long and repeated exposure, as occurs when workers are exposed to the damaged PVC material (Tuomainen et al. 2004). There is strong evidence that damaged PVC products are associated with asthma and atopic disorders (Bornehag et al. 2004; Øie et al. 1997).

The baseline exhaled NO levels were slightly higher than normal levels defined by the American Thoracic Society (1999) and the European Respiratory Society (Kharitonov et al. 1997); we believe that this is because our study group included five subjects with asthma. The baseline nasal NO levels were also higher than those reported in a previous study in the same laboratory (Stark et al. 2005). Also, two subjects had very high nasal NO values, which increased the mean levels of this small study group.

Cytokine concentrations in NAL increased after both PVC and control exposures, but we did not detect any significant differences in cytokine levels attributable to the active challenge. Consistently with unaltered nasal NO levels, the challenge with degraded PVC material did not change the NO concentrations in NAL. Moreover, the cytokine and NO levels in NAL after the vacation did not differ from the baseline levels taken before the challenges. Our timing of the NAL samplings was not entirely optimal; that is, the subjects reported a great deal of respiratory tract symptoms on the morning after PVC exposure, but NAL samples were not collected at that time.

We selected ceramic tile as the control material because it is considered a “clean” material with very low emissions. However, it is possible that exposure to ceramic tiles also evoked inflammatory changes that were seen as increased cytokine levels in NAL. Thus, another material may be more appropriate for use as control. On the other hand, it is possible that PVC exposure does not cause inflammation in upper airways that can be detected as increased cytokine or NO concentrations in NAL.

We did not detect any 2-ethylhexanol in the NAL samples collected after the PVC challenge. The compound could have evaporated from the nose before the sampling, which was performed after the lung function tests and exhaled and nasal NO measurements. It is also possible that at least a part of the compound had been absorbed into the blood. All of the exhaled breath samples collected during the PVC carpet exposure contained 2-ethylhexanol. The presence of 2-ethylhexanol in the exhaled breath is evidence that this compound is breathed into the lungs, transferred into blood, and transported to other parts of the body. It is known to be a specific, biologic marker for exposure to damaged PVC flooring material and indicates recent exposure (Wiglusz et al. 1998). In normal situations (i.e., when there is no exposure), there is no 2-ethylhexanol present in exhaled breath (Phillips et al. 1999). Although there were only 10 subjects in this study, the 2-ethylhexanol concentrations in exhaled breath correlated well with the levels in chamber air. In spite of the ventilation of the exposure chamber after PVC exposures, there were still minimal concentrations of 2-ethylhexanol in the chamber air at the time we performed the control tests. These low levels are probably not due to emissions from ceramic tiles but are probably connected to the ventilation of the exposure chamber.

In conclusion, experimental PVC material exposure evoked respiratory tract symptoms in exposed subjects but did not cause an immediate asthma-like reaction. The chamber air exposure test we developed for this study appears to be very suitable for challenge studies.

## Figures and Tables

**Table 1:** Protocol used in the chamber exposure tests for PVC and control.

Time	Protocol
Before the exposure	Exhaled and nasal NO
	Spirometry
	PEF
	Collection of NAL
Exposure (4 hr)	PEF at 1-hr intervals
	Exhaled breath sample (after 2 hr from the beginning of exposure, only in PVC exposure)
	Collection of VOC from the chamber air (sampling time 2 hr)
Immediately after the exposure	Exhaled and nasal NO
	Spirometry
	PEF
	Collection of NAL
2 hr after the exposure	Exhaled and nasal NO
	Spirometry
	PEF
Next morning after the exposure	Exhaled and nasal NO
	Spirometry
	PEF

PEF, peak expiratory flow.

**Table 2:** Exhaled NO [mean (range), parts per million] before and after exposure to PVC or control material in the exposure chamber.

Exposure	Before	Immediately after	2 hours after	Next morning
PVC	23.1 (8.0–66.7)	22.0 (6.4–61.0)	21.8 (8.3–63.0)	26.2 (6.8–68.7)
Control	23.8 (7.8–78.0)	24.9 (7.4–74.2)	25.4 (8.9–75.3)	27.1 (6.8–80.6)

**Table 3:** Nasal NO [mean (range), parts per million] before and after exposure to PVC or control material in the exposure chamber.

Exposure	Before	Immediately after	2 hours after	Next morning
PVC	151.9 (50.9–213.9)	149.9 (61.5–227.6)	148.4 (60.2–280.7)	157.9 (60.7–321.7)
Control	149.1 (47.1–227.9)	157.9 (49.9–248.8)	160.8 (51.7–279.3)	164.8 (52.0–266.3)

**Table 4:** Concentrations of cytokines TNF-α, IL-4, IL-6, and IL-12 [mean (range)] and NO [nitrite; mean (range)] in the NAL samples of the study subjects.

		PVC		Control	
Cytokine/NO	After holiday	Before	After	Before	After
TNF-α (pg/mL)	179.7 (0–1208.8)	152.2 (0–749.0)	267.2 (0–1146.8)	103.9 (0–517.6)	177.5 (0–803.0)
IL-4 (pg/mL)	51.6 (0–379.6)	56.4 (0–276.4)	102.9 (0–529.3)	39.6 (0–234.7)	80.1 (0–533.8)
IL-6 (pg/mL)	76.9 (0.2–572.4)	71.2 (0–383.9)	108.6 (0.1–536.1)	49.3 (0.5–278.4)	106.4 (0–525.9)
IL-12 (pg/mL)	308.4 (0–1863.0)	413.1 (0–2218.1)	453.2 (0–2647.8)	171.8 (0–1027.6)	435.5 (0–2650.9)
NO (μM)	6.8 (1.7–46.6)	4.8 (1.6–10.1)	6.6 (1.3–18.9)	6.1 (1.0–18.0)	4.9 (1.6–23.4)

The results are the values of samples assessed after at least a 1-week holiday and samples collected before and immediately after the challenges to PVC or control material.

## References

[b1-ehp0114-001409] American Thoracic Society (1991). Lung function testing: selection of reference values and interpretative strategies. Am Rev Respir Dis.

[b2-ehp0114-001409] American Thoracic Society (1999). Recommendations for standardized procedures for the online and offline measurement of exhaled lower respiratory nitric oxide and nasal nitric oxide in adults and children. Am J Respir Crit Care Med.

[b3-ehp0114-001409] Beasley R, Ellwood P, Asher I (2003). International patterns of the prevalence of pediatric asthma: the ISAAC program. Pedatr Clin North Am.

[b4-ehp0114-001409] Bornehag CG, Lundgren B, Weschler CJ, Sigsgaard T, Hagerhed-Engman L, Sundell J (2005a). Phthaltates in indoor dust and their association with building characteristics. Environ Health Perspect.

[b5-ehp0114-001409] Bornehag CG, Sundell J, Hagerhed-Engman L, Sigsgard T, Janson S, Aberg N (2005b). ‘Dampness’ at home and its association with airway, nose and skin symptoms among 10,851 preschool children in Sweden: a cross-sectional study. Indoor Air.

[b6-ehp0114-001409] Bornehag GC, Sundell J, Weschler CJ, Sigsgaard T, Lundgren B, Hasselgren M (2004). The association between asthma and allergic symptoms in children and phthalates in house dust: a nested case–control study. Environ Health Perspect.

[b7-ehp0114-001409] Bradford MM (1976). A rapid and sensitive method for the quantitation of microgram quantities of protein utilizing the principle of protein-dye binding. Anal Biochem.

[b8-ehp0114-001409] Green LC, Wagner DA, Glogowski J, Skipper PL, Wishnok S, Tannenbaum SR (1982). Analysis of nitrate, nitrite and [^15^N]nitrate in biological fluids. Anal Biochem.

[b9-ehp0114-001409] Hirvonen MR, Ruotsalainen M, Roponen M, Hyvärinen A, Husman T, Kosma VM (1999). Nitric oxide and pro-inflammatory cytokines in nasal lavage fluid associated with symptoms and exposure to moldy building microbes. Am J Respir Crit Care Med.

[b10-ehp0114-001409] Jaakkola JJ, Øie L, Nafstad P, Botten G, Samuelson SO, Magnus P (1999). Interior surface materials in the home and the development of bronchial obstruction in young children in Oslo, Norway. Am J Public Health.

[b11-ehp0114-001409] Jaakkola JJ, Verkasalo PK, Jaakkola N (2000). Plastic wall materials in the home and respiratory health in young children. Am J Public Health.

[b12-ehp0114-001409] Kharitonov S, Alving K, Barnes PJ (1997). Exhaled and nasal nitric oxide measurements: recommendations. Eur Respir J.

[b13-ehp0114-001409] Norbäck D, Wieslander G, Nordström K, Wålinder R (2000). Asthma symptoms in relation to measured building dampness in upper concrete floor construction, and 2-ethyl-1-hexanol in indoor air. Int J Tuberc Lung Dis.

[b14-ehp0114-001409] Øie L, Hersoug LG, Madsen JO (1997). Residential exposure to plasticizers and its possible role in the pathogenesis of asthma. Environ Health Perspect.

[b15-ehp0114-001409] Phillips M, Herrera J, Krishnan S, Zain M, Greenberg J, Cataneo RN (1999). Variation of involatile organic compound in the breath of normal humans. J Chromatogr B.

[b16-ehp0114-001409] Prat J, Xaubet A, Mullol J (1993). Immunocytologic analysis of nasal cells obtained by nasal lavage: a comparative study with a standard method of cell identification. Allergy.

[b17-ehp0114-001409] Selgrade MJK, Lemanske RF, Gilmour MI, Neas LM, Ward MDW, Henneberger PK (2006). Induction of asthma and the environment: what we know and need to know. Environ Health Perspect.

[b18-ehp0114-001409] Stark H, Randell J, Hirvonen MR, Purokivi M, Roponen M, Tukiainen H (2005). The effects of *Aspergillus fumigatus* challenge on exhaled and nasal NO levels. Eur Respir J.

[b19-ehp0114-001409] Tuomainen M, Pasanen AL, Tuomainen A, Liesvuori J, Juvonen P (2001). Usefulness of the Finnish classification of indoor climate, construction and finishing materials: comparison of indoor climate between two new blocks of flats in Finland. Atmos Environ.

[b20-ehp0114-001409] Tuomainen A, Seuri M, Sieppi A (2004). Indoor air quality and health problems associated with damp floor coverings. Int Arch Occup Environ Health.

[b21-ehp0114-001409] Viljanen AA, Halttunen PK, Kreus KE, Viljanen BC (1982). Spirometric studies in non-smoking healthy adults. Scand J Clin Lab Invest Suppl.

[b22-ehp0114-001409] Wålinder R Ernsgård L Gullstrand E Johansson G Norbäck D Venge P et al. 1999. Acute effects of experimental exposure to four volatile compounds associated with water-damaged buildings and microbial growth. In: Proceedings of Indoor Air '99, Vol.2, 8–13 August 1999, Edinburgh, Scotland. Watford, UK:Building Research Establishment Ltd, 606–611.

[b23-ehp0114-001409] Wieslander G, Norbäck D, Nordström K, Wålinder R, Venge P (1999). Nasal and ocular symptoms, tear film stability and biomarkers in nasal lavage, in relation to building-dampness and building design in hospitals. Int Arch Occup Environ Health.

[b24-ehp0114-001409] Wiglusz R, Igielska B, Sitko E, Nikel G, Jarnuszkiewicz I (1998). Emission of volatile organic compounds (VOCs) from PVC flooring coverings. Bull Inst Marit Trop Med Gdynia.

[b25-ehp0114-001409] World Medical Association 2004. Declaration of Helsinki: Ethical Principles for Medical Research Involving Human Subjects. Available: http://www.wma.net/e/policy/pdf/17c.pdf [accessed 14 July 2006].

